# Compliance to the smoke-free law in Guatemala 5-years after implementation

**DOI:** 10.1186/s12889-016-2960-x

**Published:** 2016-04-12

**Authors:** Joaquín Barnoya, Jose C. Monzon, Paulina Briz, Ana Navas-Acien

**Affiliations:** Research Department, Cardiovascular Surgery Unit of Guatemala, 9th Avenue, 8-00, Zone 11, 01011 Guatemala City, Guatemala; Division of Public Health Sciences, Department of Surgery, Washington University School of Medicine, 660 S Euclid Ave., St. Louis, MO 63110 USA; Department of Environmental Health Sciences and Institute for Global Tobacco Control, Johns Hopkins Bloomberg School of Public Health, 615 N. Wolfe Street, Baltimore, MD 21205 USA

**Keywords:** Smoke-free law, Compliance, Secondhand smoke

## Abstract

**Background:**

Smoke-free environments decrease smoking prevalence and consequently the incidence of heart disease and lung cancer. Due to issues related to poor enforcement, scant data is currently available from low/middle income countries on the long-term compliance to smoke-free laws. In 2006, high levels of secondhand smoke (SHS) were found in bars and restaurants in Guatemala City. Six months after a smoking ban was implemented in 2009, levels significantly decreased. However, in 2010, poor law compliance was observed. Therefore, we sought to assess long-term compliance to the ban using SHS measurements.

**Methods:**

In 2014 we assessed SHS exposure using airborne nicotine monitors in bars (*n* = 9) and restaurants (*n* = 12) for 7 days using the same protocol as in 2006 and in 2009. Nicotine was measured using gas-chromatography (μg/m^3^) and compared to levels pre- (2006) and post-ban (2009). Employees responded to a survey about SHS exposure, perceived economic impact of the ban and customers’ electronic cigarette use. In addition, we estimated the fines that could have been collected for each law infringement.

**Results:**

Most (71 %) venues still have a smoking section, violating the law. The percentage of samples with detectable nicotine concentrations was 100, 85 and 43 % in 2006, 2009 and 2014, respectively. In bars, median (25^th^ and 75^th^ percentiles) nicotine concentrations were 4.58 μg/m^3^ (1.71, 6.45) in 2006, 0.28 (0.17, 0.66) in 2009, and 0.59 (0.01, 1.45) in 2014. In restaurants, the corresponding medians were 0.58 μg/m^3^ (0.44, 0.71), 0.04 (0.01, 0.11), and 0.01 (0.01, 0.09). Support for the law continues to be high (88 %) among bar and restaurant employees. Most employees report no economic impact of the law and that a high proportion of customers (78 %) use e-cigarettes. A total of US$50,012 could have been collected in fines.

**Conclusions:**

Long-term compliance to the smoking ban in Guatemala is decreasing. Additional research that evaluates the determinants of non-compliance is needed and could also contribute to improve enforcement and implementation of the smoke-free law in Guatemala.

**Electronic supplementary material:**

The online version of this article (doi:10.1186/s12889-016-2960-x) contains supplementary material, which is available to authorized users.

## Background

Secondhand smoke (SHS), the mixture of mainstream and side-stream tobacco smoke, contains toxic components similar to the smoke inhaled by active smokers and is a leading cause of disease and death among children and adults worldwide [1]. According to the World Health Organization (WHO) Framework Convention on Tobacco Control (FCTC), protecting the public from SHS is key to halt the tobacco epidemic by limiting non-smokers and smokers exposure from the harmful effects of SHS [2]. Smoke-free environments decrease smoking prevalence by increasing cessation and decreasing initiation; and as a consequence, heart disease and lung cancer rates have decreased in countries and cities that have implemented smoke-free environments [1].

In 2005, Guatemala ratified the FCTC and therefore must protect citizens from SHS exposure in all workplaces [2, 3]. In 2006, a study yielded a high SHS exposure in public spaces. Exposure was highest in bars and restaurants, where nicotine concentrations were 710 and 114 times higher as compared to those found in a public hospital. Support for smoke-free environments was high (70 %) among employees [4]. In 2009, a smoke-free law was implemented, banning smoking in enclosed public places and workplaces (including bars and restaurants). In addition, the law mandates businesses to have a visible “No smoking” sign and prohibits smoking sections in all hospitality venues. The Ministry of Health is responsible for overseeing compliance and to fine businesses violating the law. Six months later, a significant reduction in SHS exposure was documented in bars and restaurants, although compliance was better in restaurants [5]. In 2010, however, an observational study reported that 86 % of bars and restaurants were non-compliant (ranging from allowing smoking indoors to inappropriate “No smoking” signage) with the law [6]. Unfortunately, smoke-free laws are faced with the challenge to ensure proper enforcement to guarantee high compliance, particularly in bars [7]. Building on previous research [4–6], we sought to provide a long-term evaluation of the law in Guatemala, a low/middle income country (LMIC) using the same methodology to assess SHS exposure and therefore allow for comparisons over time. The specific aims of our evaluation where to: 1) Examine changes in SHS levels in bars and restaurants 5 years after smoke-free law implementation, 2) Assess attitudes, knowledge, and perceived economic impact of the law in bars and restaurants, and 3) Estimate fines that would result from violations of the law.

## Methods

We assessed SHS exposure by measuring airborne nicotine concentrations in bars and restaurants in Guatemala City in 2014 (5 years after law implementation). In this cross-sectional study we used the same methodology as in 2006 and 2009 [4, 5]. Bars and restaurants were selected as they have been found to have the highest SHS exposure levels. We randomly chose different venues to avoid the risk of bias from evaluating those where we had previously measured SHS levels. SHS exposure was measured using monitors that consist of a filter cassette, a filter treated with sodium bisulfate and a nucleopore windscreen that allows airborne nicotine to reach the filter [8]. Venues were drawn from zones (the city is divided into 22 zones) 1, 10, 11, 15, and 16, where the most popular bars and restaurants are located using the same strategy previously utilized [4, 5]. All those opened for business at the time were eligible to participate. Recruitment was performed on a convenience basis, by walking through these neighborhoods and asking for permission to conduct the study. As previously done, bars and restaurants names were not recorded. After obtaining permission, two nicotine monitors were placed in different locations of the venue (e.g. cashier, table, sitting area, bar). If there was a smoking section (violating the law) monitors were placed in both smoking and non-smoking sections. Number of windows, doors, and mechanical ventilation and/or air conditioning systems for each venue were also recorded (Additional file 1). Room volume (m^3^) was obtained by estimating height, width and length. In total, 18 monitors were placed in 9 bars (1 monitor lost/stolen) and 24 in 12 restaurants (1 monitor lost/stolen) for 7 calendar days. For quality control, 10 % of samplers were duplicates and/or blanks.

At the end of the sampling period, monitors were securely closed and shipped to the Exposure Assessment Laboratory of the Institute for Global Tobacco Control at the Johns Hopkins Bloomberg School of Public Health. The filter was then extracted and analyzed via gas chromatography with nitrogen-selective detection. The intra-class correlation coefficient between duplicate samples was 0.97. Blanks comprised monitors that were opened and immediately closed afterwards in a smoke-free environment and were used to determine the blank-corrected nicotine concentrations and the nicotine limit of detection (0.013 μg/m^3^). For samples below the limit of detection, a value of half the limit of detection was assigned.

After placing the monitors, the owner or manager and one randomly selected employee were invited to answer a questionnaire (adapted from Stillman et al. [9] Additional file 2) about attitudes, knowledge, and perceived economic impact of the law. For self-reported SHS levels, the questionnaire was previously validated against hair nicotine concentrations [10]. We also collected information regarding cigarette sales, advertising or promotions from tobacco companies, and customers using electronic cigarettes (e-cigs) at the bar or restaurant.

Airborne nicotine concentration (μg/m^3^) was calculated dividing the amount of nicotine collected (μg) by the estimated volume of air that was filtered through the filter (m^3^). Nicotine concentration medians, interquartile ranges (IQR) listing the 25th and 75th percentiles, and geometric means were used to describe the data, and the Kruskal-Wallis non-parametric test was performed to compare medians across years. We first compared all three measurements and then only 2009 and 2014. In 2006, 19 monitors were placed in 10 venues (5 bars and 5 restaurants) and in 2009, 40 monitors were placed in 21 venues (10 bars and 11 restaurants) [4, 5].

We estimated the fines that could have been collected for each venue with detectable nicotine inside (smoking indoors), had a smoking section, and missing “No smoking” signage. Fines for each infringement are 100, 200, and 150, times the daily minimum wage ($10.32) [11].

Analysis was conducted using IBM SPSS Statistics for Windows Version 22.0. Institutional Review Board approval for this study was received from Francisco Marroquín University in Guatemala City.

## Results

### Changes in SHS levels in bars and restaurants 5 years after law implementation

The percentage of monitors with detectable nicotine concentrations was 43 % in 2014 (i.e. 5 years after law implementation), compared to 100 and 85 % in 2006 (i.e. before law implementation) and 2009 (i.e. 6 months after law implementation), respectively. In bars, median nicotine concentration was higher than in 2009 but lower than in 2006 (Table 1).

**Table 1:** Airborne nicotine concentrations (μg/m^3^) before and after the implementation of the Smoke-Free Law. Guatemala City

	Before			After						
				6 months			5 years			
	*n* ^a^	Median (Q1, Q3)	GM (95 % CI)	*n*	Median (Q1, Q3)	GM (95 % CI)	*n*	Median (Q1, Q3)	GM (95 % CI)	*p* ^b^
All	19	0.88 (0.48, 4.80)	1.31 (0.74, 2.29)	40	0.12 (0.04, 0.25)	0.09 (0.05, 0.15)	40	0.013 (0.01, 0.76)	0.073 (0.02, 0.142)	0.49
Bars	9	4.58 (1.71, 6.45)	3.02 (1.60, 5.59)	18	0.28 (0.17, 0.66)	0.32 (0.19, 0.53)	17	0.59 (0.01, 1.45)	0.232 (<0.01, 0.60)	0.84
Restaurants	10	0.58 (0.44, 0.71)	0.56 (0.32, 1.01)	22	0.04 (0.01, 0.11)	0.03 (0.02, 0.06)	23	0.013 (0.01, 0.09)	0.03 (0.03, 0.06)	0.50

*Q1, Q3* 25^th^ and 75^th^ percentiles, *CI* confidence interval, *GM* geometric mean, ^a^Number of monitors, ^b^ *p* values using Kruskal-Wallis test between the last two airborne nicotine measurements

In restaurants, nicotine concentrations in 2014 were the lowest of all (Table 1). In addition, consistent with previous years, the highest single nicotine level was found in a bar (10.24 μg/m^3^).

Most venues (71 %) still had a smoking section. Overall, 24 % did not have a “No smoking sign”, 62 % sold cigarettes, and 57 % had tobacco advertising.

### Attitudes, knowledge, and perceived economic impact of the smoke-free law in bars and restaurants

In total, 41 subjects completed the questionnaire (21 owners/managers and 20 employees). Respondents mean age (standard deviation) was 31 (8) years and 80 % were male. More than half (58 %) had ever smoked, 26 % reported smoking daily, 15 % less than daily, and 7 % less than weekly.

In 2006, 57 % of respondents agreed that SHS harms others, as opposed to 2009 and 2014, when all agreed. Support for the law significantly (*p* = 0.01) increased after 5 years of implementation (63 and 88 % in 2009 and 2014, respectively). When asked whether their workplace should be smoke-free, there was no difference in support in 2014 compared to 2009 (83 % vs. 82 %, respectively, *p* = 0.85) as opposed to the more than double increase from 2006 to 2009 (32 %). Regarding knowledge about the law, in 2014, 19.5 % of workers erroneously reported that the law excludes bars and restaurants. Most (90 %) perceived that the law has not had any negative economic impact and reported customers using e-cigs (78 %).

### Fines resulting from violations of the smoke-free law

Regarding fines, there were 11 venues with smoking indoors (detectable nicotine levels), 15 with smoking sections, and 5 lack a “No smoking” sign. This would account for $50,012 collected by supervising 9 bars and 12 restaurants during a 2-month period.

## Discussion

According to our findings, smoke-free law compliance in Guatemala has decreased in bars over the course of 5 years, compared to what was previously reported. Even though not statistically significant, exposure to SHS in bars has nearly doubled compared to levels 6-months after the ban was implemented. Exposure in restaurants continues to decrease, showing an improvement of the implementation in those venues, although most restaurants still have smoking sections, which is a violation of the law. Therefore, even though law implementation was successful, as a result of multiple factors (e.g., weak enforcement, poor compliance) the law has yet to become a norm.

Along with Uruguay, Panama and Mexico, Guatemala pioneered the smoke-free movement in Latin America by implementing a comprehensive law. However, long-term compliance has only been evaluated in high-income countries [12–19]. From these experiences, sustained compliance can be attained through continuous education, monitoring and enforcement [1, 13, 15]. For example in France, where an educational campaign was held prior to banning smoking in hospitality venues, there was still a sustained decrease in smoking in bars and restaurants 4 years after law implementation [18]. Guatemala, on the other hand, only did a brief educational campaign before the law was implemented. According to the Ministry of Health, there are no earmarked economic resources and only 2 inspectors are assigned for law enforcement (nationwide) and they have 16 other environmental health standards to enforce [20]. However, the weak law compliance that we found in bars is likely due to lack of political will and not economic resources, as enforcement generates revenue (at least $50,000).

Employees’ support for the law continues to improve despite poor compliance. This is not surprising as has been reported elsewhere [13, 18, 21–24]. For example, in China, even though most of the population continues to be exposed to SHS, support for smoke-free laws has increased over time [10]. Therefore, our findings are likely due to an under-resourced enforcing agency (Ministry of Health) as opposed to lack of support from hospitality venue owners/managers and workers. This calls for a strong and continuous socialization campaign to promote the law, which should include bar and restaurant owners and employees and public education through mass media. In addition, most employees reported seeing a customer smoking an e-cig. Therefore, further research is needed to determine if e-cigs are being used to circumvent the law. The impact of e-cigs as a source of occupational exposure to environmental chemicals also needs to be investigated.

Our study has several strengths and limitations. To our knowledge, this is the first study to evaluate long-term (5 years) compliance of a smoke-free law in a LMIC using the same methodology over time [4, 5]. To reduce selection bias, surveyed venues were randomly chosen without prior knowledge of compliance or notification that SHS exposure would be assessed. However, our findings should be interpreted in light of certain limitations. This is not a longitudinal assessment, but rather three cross-sectional measurements of bars and restaurants recruited in the same neighborhoods, using the same approach. Trends can only be evaluated in the context of comparing cross-sectional surveys, with the limitations that exposure levels in the venues could have changed for other reasons beyond temporal trends. We implemented the same protocol for the fieldwork but we cannot dismiss the fact that that some of the differences observed could be related to differences in performing the observation. In that situation, however, the fact that nicotine concentration increased in bars but decreased in restaurants would be harder to conclude. Our sample is not representative of the city or country and therefore the generalizability of our results is limited. Regardless, this should not affect the internal validity. Although the sample size is relatively small, it was informed by previous sample size calculations conducted for a large multi-country study [10] and it was doubled in 2009 and 2014 to further increase the power to detect differences between bars and restaurants as well as over time compared to previous surveys. Nicotine measurements were made on a continuous basis and not during the time of occupancy only. Consequently, nicotine concentrations are likely to underestimate the actual concentrations to which occupants are exposed during business hours.

## Conclusion

Although support for the smoke-free law continues to be high, compliance has been decreasing, especially in bars. Policy makers and tobacco control advocates need to revise the law to include e-cigs as they are now being used to circumvent the law. The fact that owners/managers and employees claim not to be familiar with the law deserves attention by tobacco control advocates and government authorities who should engage in educational campaigns and rigorous law enforcement.

## Ethics

Our study was approved by Francisco Marroquin University Institutional Review Board.

## Consent

An informed consent was approved and signed by every bar owner/manager and employee before answering the questionnaire.

## Additional files

Additional file 1: Nicotine monitors data collection form. (PDF 88 kb) Additional file 2: Employee SHS questionnaire. (PDF 146 kb)

## Abbreviations

SHS: secondhand smoke
e-cigs: electronic cigarettes
FCTC: framework convention on tobacco control
LMIC: low/middle income country
IQR: interquartile ranges
CI: confidence interval
GM: geometric mean
