# Supplementary material for: Compliance to the smoke-free law in Guatemala 5-years after implementation
Source: BMC Public Health. 2016 Apr 12;16:318. doi: 10.1186/s12889-016-2960-x (PMC4852414; doi:10.1186/s12889-016-2960-x)
Supplement: Additional file 1: — Nicotine monitors data collection form. (PDF 88 kb) [file 12889_2016_2960_MOESM1_ESM.pdf]

SECONDHAND SMOKE EXPOSURE AMONG BARS AND NIGHTCLUB EMPLOYEES (BAR QUESTIONNAIRE)

General Instructions:
- ***Shaded areas are to be filled by the interviewer***
- ***Read all questions, unless specified by a skip pattern***
- ***Read all options for each question. Do not read the options "decline to answer" and "Don't know/Not sure" and avoid using these options as much as possible.***
- ***Mark only one option per question, unless specified***

BAR.ID **Bar ID: ___ - __ __ - __ __**
Region Country Bar

BAR.IID **Interviewer ID: ___ - __ __ - __ __**

BAR.DATE **Date Completed: __ __ - __ __ - __ __**
D D M M Y Y

BAR.TIME **Time of Day: __ __:__ __ (24 h format)**

BAR.STS BAR STATUS: □ Smoking *1* □ Non-Smoking *2*

We would like to ask you some basic questions about the bar / nightclub. Please let me know if you do not understand any of the questions. Please answer as best as you can.

A. Key Informant Information

KII.EQX **1. Did the key informant fill out the employee questionnaire?**
Yes………………1
No………………..2

EMP.ID **If yes, please write down the subject ID from the employee questionnaire:**
___ - __ __ - __ __ - __ __
Region Country Bar Individual

KII.JOB **2. Which is your Job title in the bar / nightclub? (If more than one position, mark the highest position)**
Owner……………………………………… 1
Manager……………………………………. 2
Bartender (including bar back)…………….. 3
Waiter (including other table services)…….. 4
Cook (including other kitchen jobs) ……….. 5
Other ………………………………………. 6 *KII.JOO* (Specify) ___________________________
Decline to answer………………………….. 9

B. Bar / Night Club Characteristics

I'd like to ask you some questions about the characteristics of the bar / nightclub.

BNC.YRS **3. How many years has been the bar / nightclub in business?** ________ (in years)
Don't know / not sure……………………… 777

BNC.NEI **4. What is the name of the area/ neighborhood in which the bar/nightclub is located?**

Area/ Neighborhood: ________________________

BNC.SZE **5. What is the size of the indoor bar area open to customers? ________** (square meters)
Don't know / not sure……………………… 777

BNC.OCP **6. What is the maximum occupancy of the bar/nightclub? (Occupancy that is legally allowed)**
Total: _________
Don't know / not sure……………………… 777

7. What are the hours of operation of the bar / nightclub (24h format)?

	MON	TUES	WED	THURS	FRI	SAT	SUN
Not open	*BNC.HMO* □ DK 99	*BNC.HTU* □ DK 99	*BNC.HWE* □ DK 99	*BNC.HTH* □ DK 99	*BNC.HFR* □ DK 99	*BNC.HSA* □ DK 99	*BNC.HSU* □ DK 99
Open (time)	*BNC.OT1* _ _:_ _	*BNC.OT2* _ _:_ _	*BNC.OT3* _ _:_ _	*BNC.OT4* _ _:_ _	*BNC.OT5* _ _:_ _	*BNC.OT6* _ _:_ _	*BNC.OT7* _ _:_ _
Close (time)	*BNC.CT1* _ _:_ _	*BNC.CT2* _ _:_ _	*BNC.CT3* _ _:_ _	*BNC.CT4* _ _:_ _	*BNC.CT5* _ _:_ _	*BNC.CT6* _ _:_ _	*BNC.CT7* _ _:_ _
Total hours open	*BNC.TMO*	*BNC.TTU*	*BNC.TWE*	*BNC.TTH*	*BNC.TFR*	*BNC.TSA*	*BNC.TSU*

BNC.EMP **8. How many employees work in this bar / nightclub? ____________** employees
Don't know / not sure……………………… 777

BNC.ECS **9. Do you know how many of the employees currently smoke?**
Yes…………………………………………. 1 *BNC.EHM* How many:___________
No………………………………………….. 2
Don't know / not sure……………………… 777

BNC.CWD **10. How many customers are served on an average *weekday* night (Sunday – Thursday)? __________**
Don't know / not sure……………………… 777

BNC.CWN **11. How many customers are served on an average *weekend* night (Friday – Saturday)? __________**
Don't know / not sure……………………… 777

BNC.AGE **12. What is your estimate of the average age of the customers?**
<18…………………………………………. 1
18-24……………………………………….. 2
25-29……………………………………….. 3
30-39……………………………………….. 4
40 and above……………………………….. 5
Mixed/all ages………………………………6
Don't know / not sure……………………… 777

BNC.EDU **13. What is your estimate of the average education level of the customers?**
Primary school or less………………………1
High school education………………………2
College/University education……………… 3
Mixture of education levels…………………4
Don't know / not sure……………………….7

BNC.LOC **14. Where are the <u>usual</u> customers of the bar / nightclub from?**

Local/neighborhood crowd………………… 1
Customers from all over the city…. ………. 2
Tourist/out-of-towners……………………3
Mixed crowd…………………………….. 4
Don't know / not sure…………………… 7

BNC.CCS **15. What is your estimate of the percentage of customers who smoke inside of the bar/nightclub?**

None……………………………………... 1
1 to 24%...2
25 to 50%......................................………..3
50 to 74%...4
75% or more……………………………...5
Don't know………………………………7

BNC.VEN **16. Does the bar/nightclub have a ventilation system for extracting smoke?**

Yes………………………………………. 1
No……………………………………….. 2
Don't know / not sure…………………… 7

BNC.VON **17. If yes, is the ventilation system on during opening hours?**

Yes………………………………………. 1
No……………………………………….. 2
Sometimes………..……………………3

BNC.ACS **18. Does the bar/nightclub have an air conditioning system?**

Yes………………………………………. 1
No……………………………………….. 2
Don't know / not sure…………………… 7

BNC.AON **19. If yes, is the air conditioning system on during opening hours?**

Yes………………………………………. 1
No……………………………………….. 2
Sometimes………..……………………3

BNC.OUT **20. Does the bar / nightclub have an outdoor area (terrace, patio, other)?**

Yes………………………………………. 1
No……………………………………….. 2

BNC.FOD **21. Does the bar / nightclub serve food?**

Yes, full menu…………………………... 1
Yes, only small plates………… ……………2
No…………………………….………….. 3

BNC.DAN **22. Is there space designated for dancing?**

Yes………………………………………. 1
No……………………………………….. 2

BNC.MUS **23. Does the bar/nightclub offer live music (at any time during the week)?**

Yes………………………………………. 1
No……………………………………….. 2

C. Smoking Policy

Now I'd like to ask you some questions about smoking policies of the bar/nightclub.

POL.POL **24. Does this establishment have a policy that restricts smoking in any way?**

Yes…………………………………………. 1
No………………………………………….. 2
Don't know / not sure……………………… 7

BNC.WRI **25. If yes, is the smoking policy in a written format?**

Yes…………………………………………. 1
No………………………………………….. 2
Don't know / not sure……………………… 7

POL.IND **26. Please specify which of these best describes this bar / nightclub smoking policy for <u>indoor</u> areas**

Not allowed in any indoor areas…………… 1 → 26.1
Allowed in some indoor areas …………….. 2 → 26.2 and 26.3
Allowed in all indoor areas ………………...3 → 26.2 and 26.3
Don't know/ not sure………………………. 7

POL.INF ***26.1 If smoking is not allowed in any indoor area*, is this policy strictly enforced?**

Yes…………………………………………. 1
No………………………………………….. 2
Don't know / not sure……………………… 7

POL.REA ***26.2 If smoking is allowed*, what are the main reasons?**

Customers preference……………………… 1
Concern over lost profits……………………2
Both………………………………………… 3
Other ………………………………………. 4 *POL.REO* Specify: ______________________________
Don't know / not sure……………………….7
Decline to answer…………………………...9

POL.VOL ***26.3. If smoking is allowed*, how likely is the bar/nightclub to go <u>voluntarily</u> smoke-free in the next 12 months?**

Very likely…………………………………..1
Somewhat likely…………………………… 2
Somewhat unlikely………………………….3
Very unlikely………………………………..4
Don't know / not sure……………………….7
Decline to answer………………………….. 9

POL.SGN **27. Are there "No Smoking" signs posted inside the bar/nightclub?**

Yes…………………………………………. 1
No………………………………………….. 2

POL.CIG **28. Are cigarettes sold anywhere inside the bar/nightclub?**

Yes…………………………………………. 1
No………………………………………….. 2

CIG.SLD **28*.1 If yes, how are cigarettes sold?***

Vending machines…………………………..1
Over the bar counter………………………...2
Outside vendors……………………………..3
Other………………………………………...4
Don't know / not sure……………………… 7

POL.TOB **29. Is there tobacco advertising in the bar/nightclub?**

Yes…………………………………………. 1
No………………………………………….. 2

TOB.ADV **29.1 If yes, please describe**: ____

____

POL.PRO **30. Do you receive free promotional items from tobacco companies?**

Yes…………………………………………. 1
No………………………………………….. 2

PRO.TOB **30.1. If yes, please describe**: ____

____

EMP.PRE **31. Now, as a last question, how many employees are present now?**

______________ employees

Don't know ………………….777

This is the end of the bar / nightclub questionnaire. Thank you very much for your collaboration.

AIR SAMPLE MONITORING SAMPLE SHEET (To be filled by the field worker)

In each bar, 2 monitors must be placed for a 1 week period. If the bar has a smoking and a non-smoking area, place one monitor in each area. If the bar has two or more different spaces or rooms, you can place monitor 1 in one room and monitor 2 in another room. If the bar has only one space or room and there is no distinction between smoking-non-smoking areas, place each monitor in two different areas within the space, such as one monitor in the bar area and the other monitor in the tables area.

AIR.AM1 **Air Monitor 1 ID: ___ - __ __ - __ __ - __ __ __ __**<br>Region Country Bar Lab ID

AIR.DUP Does a duplicate air monitor accompany air monitor 1 (only 1 duplicate every 10 air monitors 1 or 2 placed)?

- □ Yes *1* *AIR.DID* If yes, please indicate Duplicate Air Monitor ID: ___ - __ __ - __ __ - __ __ __ __
- □ No *2*

Region Country Bar Lab ID

AIR.BLK Does a blank air monitor accompany air monitor 1 (only 1 blank every 10 air monitors 1 or 2 placed)?

- □ Yes *1* *AIR.BID* If yes, please indicate Blank Air Monitor ID: ___ - __ __ - __ __ - __ __ __ __
- □ No *2*

Region Country Bar Lab ID

AIR.DP1 Date of placement: __ __ /__ __ / __ __ *AIR.TP1* Time: ____: ____ (24h)

AIR.DV1 Date of visit: __ __ /__ __ / __ __ *AIR.TV1* Time: ____: ____ (24h)

AIR.DR1 Date of removal: __ __ /__ __ / __ __ *AIR.TR1* Time: ____: ____ (24h)

AIR.PL1 Place within the bar or night club: ____________________________________

Room dimensions: *AIR.RL1*Length: _____ x *AIR.RW1*Width _____ x *AIR.RH1* Height _____ (meters)
AIR.RD1 Volume: _____ m^3

AIR.SA1 Is smoking allowed in this room/area?

Yes………………………………………….1
No………………………………………….. 2

AIR.ND1 Number of doors open to outdoor areas: _____________

AIR.MD1 Most doors are:

Hollow or always open…………………….. 1
Cloth, bamboo, other soft material……….. 2
Wood, glass, other hard material………….. 3

AIR.NW1 Number of windows open to outdoor areas: __________

AIR.MW1 Most windows are:

Hollow or always open…………………….. 1
Cloth, bamboo, other soft material……….. 2
Wood, glass, other hard material………….. 3

OPN.PT1 Overall, what percent of the establishment walls are generally open to outdoor air through doors, windows and other open spaces?

0%.. 1
0 to 9%... 2
10 to 24%... 3
24 to 49%... 4
50% or more……………………………...5

		Placement	Visit day	Removal
		AIR.DOP1	*AIR.DOV1*	*AIR.DOR1*
Are the doors open?	*Circle:*	Yes *1*/No *2*	Yes *1*/No *2*	Yes *1*/ No *2*
		AIR.WOP1	*AIR.WOV1*	*AIR.WOR1*
Are the windows open?		Yes *1*/No *2*	Yes *1*/No *2*	Yes *1*/ No *2*
		AIR.VSP1	*AIR.VSV1*	*AIR.VSR1*
Is a ventilation system or AC on?		Yes *1*/No *2*	Yes *1*/No *2*	Yes *1*/ No *2*
			AIR.CPV1	*AIR.CPR1*
Monitor in the correct place?			Yes *1*/No *2*	Yes *1*/No *2*
			AIR.FIV1	*AIR.FIV1*
If not in the correct place, could you find it?			Yes *1*/No *2*	Yes *1*/No *2*

If found in the incorrect location, where did you find it? *AIR.WH1* ________________________________

For visit day only (on a day and time of average occupancy):

AIR.EO1 Estimated occupancy: ___________

AIR.NS1 Number of smokers visible during a 15-minute period: ___________

AIR.MD1 Minimal distance to the filter of the nearest smoker: ___________ meters

AIR.AM2 **Air Monitor 2 ID: ___ - __ __ - __ __ - __ __ __ __**
Region Country Bar Lab ID

AIR.DP2 Date of placement: __ __ /__ __ / __ __ *AIR.TP2* Time: ____: ____ (24h)

AIR.DV2 Date of visit: __ __ /__ __ / __ __ *AIR.TV2* Time: ____: ____ (24h)

AIR.DR2 Date of removal: __ __ /__ __ / __ __ *AIR.TR2* Time: ____: ____ (24h)

AIR.PL2 Place within the bar or night club: ___________________________________

AIR.AMS Is this room the same than for Air Monitor 1?
Yes the room is the same……………………………………………………………1
No, the room is different but it is communicated with the previous room…………….2
No, the room is different and it is completely separated from the previous room…… 3

If the room is the same, this is the end of the questionnaire → fill the diagram.
If the room is different, please continue with the questionnaire:

AIR.RD2 Room dimensions: *AIR.RL1*Length: _____ x *AIR.RW1*Width _____ x *AIR.RH1* Height _____

AIR.SA2 Is smoking allowed in this room/area?
Yes…………………………………………. 1
No………………………………………….. 2

AIR.ND2 Number of doors open to outdoor areas: _____________

AIR.DM2 Most doors are:
Hollow or always open…………………….. 1
Cloth, bamboo, other soft material……….. 2
Wood, glass, other hard material………….. 3

AIR.NW2 Number of windows open to outdoor areas: __________

AIR.WM2 Most windows are:
Hollow or always open…………………….. 1
Cloth, bamboo, other soft material……….. 2
Wood, glass, other hard material………….. 3

OPN.PT2 Overall, what percent of the establishment walls are generally open to outdoor air through doors, windows and other open spaces?
0%.. 1
0 to 9%... 2
10 to 24%... 3
24 to 49%... 4
50% or more………………………………...5

		**Placement**	**Visit day**	**Removal**
		AIR.DOP2	*AIR.DOV2*	*AIR.DOR2*
Are the doors open?	*Circle:*	Yes *1*/No *2*	Yes *1*/No *2*	Yes *1*/ No *2*
		AIR.WOP2	*AIR.WOV2*	*AIR.WOR2*
Are the windows open?		Yes *1*/No *2*	Yes *1*/No *2*	Yes *1*/ No *2*

		AIR.VSP2	*AIR.VSV2*	*AIR.VSR2*
Is a ventilation system or AC on?		Yes *1*/No *2*	Yes *1*/No *2*	Yes *1*/ No *2*
Monitor in the correct place?			*AIR.CPV2* Yes *1*/No *2*	*AIR.CPR2* Yes *1*/No *2*
If not in the correct place, could you find it?			*AIR.FIV2* Yes *1*/No *2*	*AIR.FIV2* Yes *1*/No *2*

If found in the incorrect location, where did you find it? *AIR.WH2* ________________________________

For visit day only (on a day and time of average occupancy):

AIR.EO2 Estimated occupancy: ___________

AIR.NS2 Number of smokers visible during a 15-minute period: ___________

AIR.MD2 Minimal distance to the filter of the nearest smoker: ___________ meters

Diagram to indicate the location of the air monitors and of the outdoor windows and doors within the bar

Location in room of air monitors 1 and 2 (and duplicate and blanks if corresponding). Please indicate on diagram the location of the monitor and any windows and doors that open to outdoor air:

Area 1

Area 2 (If both monitors are in same room, use first box for both)

**X1, X2**	Air monitors	□ (X) normal □ (D) duplicate □ (B) blank
(red dotted line)	Door	
(green double arrow)	Window	

AIR.COB Other comments and observations related to the air samplers or bar:

____

____

____
