# Supplementary material for: Compliance to the smoke-free law in Guatemala 5-years after implementation
Source: BMC Public Health. 2016 Apr 12;16:318. doi: 10.1186/s12889-016-2960-x (PMC4852414; doi:10.1186/s12889-016-2960-x)
Supplement: Additional file 2: — Employee SHS questionnaire. (PDF 146 kb) [file 12889_2016_2960_MOESM2_ESM.pdf]

Secondhand Smoke Exposure Among Bar and Nightclub Employees (Employee Questionnaire)

General Instructions:

- ***Shaded areas are to be filled by the interviewer***
- ***Read all questions, unless specified by a skip pattern***
- ***Read all options for each question. Do not read the options "decline to answer" and "Don't know/Not sure" and avoid using these options as much as possible.***
- ***Mark only one option per question, unless specified***

EMP.ID **Employee ID: ___ - __ __ - __ __ - __ __**
Region Country Bar Employee

EMP.IID **Interviewer ID: ___ - __ __ - __ __**

EMP.DATE **Date Completed: __ __ - __ __ - __ __**
DD MM YY

EMP.TIME **Time of Day: __ __:__ __ 24hr format**

We would like to ask you some basic questions demographic questions and some questions about smoking and exposure to secondhand smoke. Please let me know if you do not understand any of the questions. Please answer as best as you can.

A. Demographic and Occupational Information

DEM.AGE **1. What is your current age?** ________ (in years)
Decline to answer …………………………..999

DEM.DOB **2. What is your date of birth?** (day/ month/year) ___ / ___ / ___
Decline to answer …………………………. 999

DEM.SEX **3. Record sex from observation, ask if necessary:**
Male ………………………………………...1
Female...2

DEM.EDU **4. How many years did you go to school?**
Less than High School …………………….. 1
High School or equivalent ………………… 2
College / University ………………………..3
Decline to answer …………………………..9

DEM.POS **5. What is your current position in this bar / nightclub? (If more than one position, mark the highest position)**
Owner……………………………………… 1
Manager…………………………………….2
Bartender (including bar back)…………….. 3
Waiter (including other table services)……..4
Cook (including other kitchen jobs) ………..5
Other ………………………………………. 6 *DEM.OPO* (Specify) ________________________________
Decline to answer………………………….. 9

DEM.EMY **6. How long have you been at your current job?** ________ years (round off to 0.25, 0.5 or 0.75 years)
Decline to answer………………………….. 99

DEM.EMD **7. How many days a week do you typically work in this establishment?** _____ (number between 1 and 7)

Decline to answer …………………………..9

DEM.EMH **8. How many hours do you work during a typical shift? _____________** hours (1 to 24, usually <16)

Decline to answer………………………….. 99

DEM.OEM **9. Are you currently employed in another job(s)?**

Yes ……………………………………….... 1 *DEM.OJO* specify other job: ________________

No…... 2

B. Smoking Behavior and Secondhand Smoke Exposure

Now, I would like to ask you about your smoking history.

SMK.EXP **10. Have you ever tried or experimented with smoking, even just one puff?**

Yes ……………………………………….... 1

No…... 2 Skip to 17

SMK.100 **11. Have you smoked at least 100 cigarettes or the equivalent in your lifetime [100 cigarettes = 5 packs]?**

Yes ……………………………………….... 1

No…... 2 Skip to 17

Don't know/ Not sure……………………… 7 Skip to 17 (avoid this option as much as possible)

SMK.TOB **12. If yes, what kind of tobacco do or did you smoke the most?**

Cigarette with filter…………………………1

Cigarette without filter……………………...2

Other forms of tobacco…………………….. 3 *SMK.TYP* Specify: ____________________________ (cigar, hand-rolled tobacco, pipe with/without water, etc.)

Don't know/ not sure………………………. 7

SMK.AGE **13. How old were you when you first started to smoke (cigarettes, pipes, cigars) regularly?**

_________ years

Declined to answer………………………… 999

Don't know/ not sure………………………. 777

SMK.1YR **14. In the past year, did you smoke tobacco on a daily basis, less than daily, or not at all?**

Daily……………………………………….1

Less than daily…..……………………….2

Not at all……………….. ………………3

SMK.QIT **15. If you quit smoking (cigarettes / pipes / cigars), how long has it been since you quit?**

_________ years

Declined to answer………………………… 999

Don't know/ not sure………………………. 777

SMK.NUM **16. On average, how many [manufactured cigarettes, hand-rolled cigarettes, cigars, pipes] <u>do / did</u> you usually smoke each day?**

__________ cigarettes / per day

Don't know/ not sure………………………. 777

SMK.SLT **17. In the past year, did you use smokeless tobacco (snuff, chewing tobacco, or dip) on a daily basis, less than daily, or not at all?**

Daily……………………………………….1

Less than daily…..……………………….2

Not at all……………….. ………………3
Don't know/ not sure……………………7

The following set of questions is related to exposure to secondhand smoke at home. By secondhand tobacco smoke we mean that you can see and / or smell other people's tobacco smoke, or that you are otherwise aware that tobacco smoke is in the air. First I would like to ask you about how many adults and children live in your home:

SHS.LIV **18. How many adults (18 years and above) live in your home, excluding yourself?**
_________ adults

Declined to answer………………………………999

SHS.LIC **19. How many children (less than 18 years) live in your home?**
_________ children

Declined to answer……………………………….999

If no adult or children live in the home, skip to 22

SHS.HMS **20. Do other household members smoke?**
Yes, at least one household member smokes..1
No, no other household member smokes……2 Skip to 23
Don't know / Not sure……………………….7 Skip to 23

SHS.NMS **21. In total, how many of your other household members smoke (excluding yourself)?**
____

22. For each household member who smokes:

	**Relationship of person to interviewee**	**What kind of tobacco does the member of the household usually smoke?**	**How many [cigarettes, cigars, pipes] does the member of the household usually smoke?**	**Do the household members smoke INSIDE THE HOME?**
Member 1	*SHS.MM1*<br>Spouse, partner…….1<br>Siblings, parents, other family…….2<br>Friend, roommates...3<br>Other……………….4	*SHS.KT1*<br>Cigarettes……………….. 1<br>Other (cigar, hand-rolled tobacco, pipe, etc.)....... 2<br>*SMK.TH1*____________<br>Don't know / Not sure ….. 7	*SHS.QS1*<br>_____ cigarettes / day<br><br>Don't know/not sure…… 777	*SHS.QH1*<br>Yes……………...1<br>No………………2<br>Don't know/Not sure ………..7
Member 2	*SHS.MM2*<br>Spouse, partner…….1<br>Siblings, parents, other family…….2<br>Friend, roommates...3<br>Other……………….4	*SHS.KT2*<br>Cigarettes……………….. 1<br>Other (cigar, hand-rolled tobacco, pipe, etc.)....... 2<br>*SMK.TH2*____________<br>Don't know / Not sure ….. 7	*SHS.QS2*<br>_____ cigarettes / day<br><br>Don't know/not sure…… 777	*SHS.QH2*<br>Yes……………...1<br>No………………2<br>Don't know/Not sure ………..7
Member 3 or more	*SHS.MM3*<br>Spouse, partner…….1<br>Siblings, parents, other family…….2<br>Friend, roommates...3<br>Other……………….4	*SHS.KT3*<br>Cigarettes……………….. 1<br>Other (cigar, hand-rolled tobacco, pipe, etc.)....... 2<br>*SMK.TH3*____________<br>Don't know / Not sure ….. 7	*SHS.QS3*<br>_____ cigarettes / day<br><br>Don't know/not sure…… 777	*SHS.QH3*<br>Yes……………...1<br>No………………2<br>Don't know/Not sure ………..7

SHS.POL **23. Does your home have rules that restrict smoking indoors in any way?**
Yes ……………………………………….... 1
No…... 2 Skip to 24

Don’t know/ Not sure……………………… 7 Skip to 24 (avoid this option as much as possible)

SHS.SMK **23.1. If yes, please specify your home’s indoor smoking rules:**

Not allowed in any indoor areas…………… 1
Allowed in some indoor areas………………2 Skip to 24
Allowed in all indoor areas…………………3 Skip to 24

SHS.SKP **23.2. If smoking is not allowed in any indoor areas, how long has this rule been in place?**

________ years

SHS.ENF **23.3. If smoking is not allowed in any indoor area, is this rule strictly enforced?**

Yes ……………………………………….... 1
No…... 2
Don’t know/ Not sure……………………… 7

The next set of questions I am going to ask you about exposure to secondhand tobacco smoke in general, including exposure at work:

SHS.DDS **24. How many days per week are you usually exposed to secondhand smoke (including work days)?**

________ (number between 0 and 7)
Decline to answer …………………………..9

SHS.HRS **25. How many hours per day are you usually exposed to secondhand smoke (including work days)?**

________ (number between 0 and 24, usually less than 16)
Decline to answer …………………………..99

SHS.SML **26. When you go home from work do you have a residual smell of cigarette smoke on your clothes or hair?**

Yes ……………………………………….... 1
No…... 2
Don’t know/ Not sure……………………… 7

C. Employee Health

Now, I would like to ask you some questions about your health.

HEA.ATH **27. Has a doctor or other health professional ever told you that you had asthma?**

Yes ……………………………………….... 1
No…... 2
Don’t know/ Not sure……………………… 7

HEA.RES **28. Has a doctor or other health professional ever told you that you had any other respiratory disease?**

Yes ……………………………………….... 1 *HEA.ORD* Specify: ___________________________
No…... 2
Don’t know/ Not sure……………………… 7

29. Have you experienced any of the following sensory symptoms in the past 4 weeks?

HEA.RED **29.1. Red or irritated eyes**

Yes ……………………………………….... 1
No…... 2
Don’t know/ Not sure……………………… 7

HEA.RUN **29.2. Runny nose, sneezing, or nose irritation**
Yes ………………………………………..... 1
No….. 2
Don't know/ Not sure……………………… 7

HEA.SOR **29.3. Sore or scratchy throat**
Yes ………………………………………..... 1
No….. 2
Don't know/ Not sure……………………… 7

30. Have you experienced any of the following respiratory symptoms in the past 4 weeks?

HEA.WHE **30.1. Wheezing or whistling in chest**
Yes ………………………………………..... 1
No….. 2
Don't know/ Not sure……………………… 7

HEA.SOB **30.2. Feeling short of breath**
Yes ………………………………………..... 1
No….. 2
Don't know/ Not sure……………………… 7

HEA.CAM **30.3. Coughing in morning**
Yes ………………………………………..... 1
No….. 2
Don't know/ Not sure……………………… 7

HEA.CPM **30.4. Coughing during the rest of the day or at night**
Yes ………………………………………..... 1
No….. 2
Don't know/ Not sure……………………… 7

HEA.PHL **30.5. Bringing up any phlegm**
Yes ………………………………………..... 1
No….. 2
Don't know/ Not sure……………………… 7

31. To what extent have you experienced the following over the past 4 weeks:

0 = Absent 1 = Mild 2 = Moderate 3 = Severe

HEA.DPM	**Depressed mood**	**0**	**1**	**2**	**3**
HEA.ISM	**Insomnia**	**0**	**1**	**2**	**3**
HEA.IFA	**Irritability, frustration, or anger**	**0**	**1**	**2**	**3**
HEA.ANX	**Anxiety**	**0**	**1**	**2**	**3**
HEA.DCO	**Difficulty concentrating**	**0**	**1**	**2**	**3**
HEA.RLN	**Restlessness**	**0**	**1**	**2**	**3**
HEA.EAT	**Increased appetite or weight gain**	**0**	**1**	**2**	**3**

D. Opinion

Finally, I will ask you a few opinion questions.

OPI.BAN **32. Do you think that bars and nightclubs should be smoke-free (smoking not allowed in any indoor areas)?**

Yes (smoking should not be allowed in any indoor areas)…………..…................ 1
No (smoking should be allowed in some or all indoor areas)............................. 2
Does not matter……………………………..………………………………………. 3
Declined to answer…………………………………………………………………9

OPI.SFE **33. Do you prefer to work in a smoke-free establishment?**

Yes ……………………………………….... 1
No…... 2
Does not matter…………………………….. 3
Declined to answer………………………….9

OPI.RSN **If yes, which is your main reason?**

Health concerns……………………………..1
Better air quality……………………………2
Both………………………………………...3
Other………………………………………..4 *OPI.OTH* Specify: _____________________
Don't know / not sure………………………7

If non-smoker, skip 35 and 36

OPI.LAC **34. *For current smokers*: Do you think that a smoke-free law will help you to quit smoking?**

Yes ……………………………………….... 1
No…... 2
Don't know / not sure ….………………….. 7
Declined to answer………………………….9

OPI.LAP **35. *For former smokers*: Do you think that a smoke-free law will help you to remain a non-smoker?**

Yes ……………………………………….... 1
No…... 2
Don't know / not sure ….………………….. 7
Declined to answer………………………….9

Interview is over. Now we are going to proceed to the hair collection. While I get ready, I would like to ask you to fill the following form about exposure to secondhand smoke in different locations. Let me know if you need any help to fill the form. [give the form in the next page to the employee and get ready to collect the hair sample]

FREQUENCY OF EXPOSURE TO SECONDHAND SMOKE – SELF-REPORTED FORM if interviewed based not possible

Employee ID: ___ - __ __ - __ __ - __ __ ***Confirm this matches Subject ID on page 1*

Region Country Bar Employee

Please tell us about any secondhand smoke exposure you may have had during a typical weekday and weekend in each of the locations listed below.

(If you spend 0 hours per day in a location, please check "Not Applicable")

Think about a typical <u>weekday</u> (Sunday through Thursday) over the past 30 days:

**In each of the following places**	**How many hours per day do you spend in each of these places?**	**Were you exposed to secondhand smoke?**				
		**Always** *1*	**Often** *2*	**Rarely/ Sometimes** *3*	**Never** *4*	**Not Applicable** *99*
At work	*SHS.HW1* ______hours per day	*SHS.HW2* ◻	◻	◻	◻	◻
Home or home of others	*SHS.HH1* ______hours per day	*SHS.HH2* ◻	◻	◻	◻	◻
Transportation vehicle	*SHS.HT1* ______hours per day	*SHS.HT2* ◻	◻	◻	◻	◻
Restaurant <u>(other than workplace)</u>	*SHS.HR1* ______hours per day	*SHS.HR2* ◻	◻	◻	◻	◻
Bar (<u>other than workplace</u>)	*SHS.HA1* ______hours per day	*SHS.HA2* ◻	◻	◻	◻	◻
Other public places*	*SHS.HP1* ______hours per day	*SHS.HP2* ◻	◻	◻	◻	◻
Out in the open	*SHS.HO1* ______hours per day	*SHS.HO2* ◻	◻	◻	◻	◻

*Other public places include shopping places, government public places, educational facilities, religious facilities, sport facilities, waiting rooms, train / bus stations, etc.

Think about a typical <u>weekend</u> day (Friday and Saturday) over the past 30 days:

**In each of the following places**	**How many hours per day do you spend in each of these places?**	**Were you exposed to secondhand smoke?**				
		**Always** *1*	**Often** *2*	**Rarely/ Sometimes** *3*	**Never** *4*	**Not Applicable** *99*
At work	*SHS.HW3* ______hours per day	*SHS.HW4* ◻	◻	◻	◻	◻

		SHS.HH4				
Home or home of others	*SHS.HH3*<br>______hours per day	□	□	□	□	□
		SHS.HT4				
Transportation vehicle	*SHS.HT3*<br>______hours per day	□	□	□	□	□
		SHS.HR4				
Restaurant (other than workplace)	*SHS.HR3*<br>______hours per day	□	□	□	□	□
		SHS.HA4				
Bar (other than workplace)	*SHS.HA3*<br>______hours per day	□	□	□	□	□
		SHS.HP4				
Other public places*	*SHS.HP3*<br>______hours per day	□	□	□	□	□
		SHS.HO4				
Out in the open	*SHS.HO3*<br>______hours per day	□	□	□	□	□

*Other public places include shopping places, government public places, educational facilities, religious facilities, sport facilities, waiting rooms, train / bus stations, etc.

PLEASE RETURN TO INTERVIEWER WHEN FINISHED.
THANK YOU!

HAIR SAMPLING

HSS.ID Hair ID: ___ - __ __ - __ __ - __ __ ***Confirm this matches Subject ID on page 1*

HSS.DATE Date of sampling: __ __ /__ __ / __ __ *HSS.TIME* Time: __ __: __ __ (24h format)
DD MM YY

Before collecting the hair, I would like to ask you a couple of questions:

HSS.CTR **H.1. Has the hair had any chemical treatment (color or dye, bleach, highlighted, perm or straightened)?**

Yes ………………………………………... 1
No….. 2 Skip to H.2
Don’t know / not sure ….………………….. 7 Skip to H.2
Declined to answer………………………….9 Skip to H.2

HSS.AGO If yes, what and how long ago was the last treatment? __________

HSS.CTR Type of chemical treatment: __________________________________

HSS.DCT Date of chemical treatment: __ __ /__ __ / __ __
DD MM YY

SMK.30D **H.2. In the past 30 days, did you smoke on a daily basis, less than daily, a few puffs or not at all?**

Daily…..……………………………………..1
Less than daily…………………………….. 2
Only a few puffs…………….…….……….. 3
Not smoked at all …………..……………… 4

NRT.30D **H.3 In the past 30 days, have you ever used nicotine replacement therapy (gum, patch, tablets)?**

Yes …………………………………..... 1
No….. 2
Don’t know / Not sure ………………………7

HSS.OBT **H.4 To fill by the interviewer: was the hair sample successfully obtained?:**

Yes …………………………………..... 1
No….. 2

HSS.NOT **If No, why not?:**

Hair too short / no hair…………..……..... 1
Participant refused providing the sample...... 2
Other………………………………………… 3 *HSS.OTH* Specify: ______________________________

HSS.COL **If Yes, what color is the hair?:**

Black……………..……..... 1
Brown…………………........ 2
Blond.…………………........ 3
Grey...……………………....... 3 [>10% grey hair]
White…………………….. 4 [>80% white hair, otherwise select grey]
Other……………………… 5 *HSS.COL,OTH* Specify: ______________________________

HSS.TYP **If Yes, what type is the hair?:**

Straight………..……..... 1
Wavy.…………………........ 2
Curly.…………………........ 3

HSS.COB Other comments and observations related to the hair collection or to the questionnaire overall:

____

____

____

____

____

____

____
